# Lipid tethering of breast tumor cells enables real-time imaging of free-floating cell dynamics and drug response

**DOI:** 10.18632/oncotarget.7251

**Published:** 2016-02-08

**Authors:** Kristi R. Chakrabarti, James I. Andorko, Rebecca A. Whipple, Peipei Zhang, Elisabeth L. Sooklal, Stuart S. Martin, Christopher M. Jewell

**Affiliations:** ^1^ Medical Scientist Training Program, University of Maryland School of Medicine, Baltimore, MD 21201, USA; ^2^ Graduate Program in Molecular Medicine, University of Maryland School of Medicine, Baltimore, MD 21201, USA; ^3^ Marlene and Stewart Greenebaum Cancer Center, University of Maryland School of Medicine, Baltimore, MD 21201, USA; ^4^ Fischell Department of Bioengineering, University of Maryland, College Park, MD 20742, USA; ^5^ Department of Physiology, University of Maryland School of Medicine, Baltimore, MD 21201, USA; ^6^ Department of Microbiology and Immunology, University of Maryland School of Medicine, Baltimore, MD 21201, USA

**Keywords:** circulating tumor cells, microtentacles, breast cancer, microfluidics, polyelectrolyte multilayers

## Abstract

Free-floating tumor cells located in the blood of cancer patients, known as circulating tumor cells (CTCs), have become key targets for studying metastasis. However, effective strategies to study the free-floating behavior of tumor cells *in vitro* have been a major barrier limiting the understanding of the functional properties of CTCs. Upon extracellular-matrix (ECM) detachment, breast tumor cells form tubulin-based protrusions known as microtentacles (McTNs) that play a role in the aggregation and re-attachment of tumor cells to increase their metastatic efficiency. In this study, we have designed a strategy to spatially immobilize ECM-detached tumor cells while maintaining their free-floating character. We use polyelectrolyte multilayers deposited on microfluidic substrates to prevent tumor cell adhesion and the addition of lipid moieties to tether tumor cells to these surfaces through interactions with the cell membranes. This coating remains optically clear, allowing capture of high-resolution images and videos of McTNs on viable free-floating cells. In addition, we show that tethering allows for the real-time analysis of McTN dynamics on individual tumor cells and in response to tubulin-targeting drugs. The ability to image detached tumor cells can vastly enhance our understanding of CTCs under conditions that better recapitulate the microenvironments they encounter during metastasis.

## INTRODUCTION

Cancer metastasis occurs when epithelial tumor cells travel through non-adherent microenvironments, like the bloodstream or lymphatics, to a distant organ. The presence of tumor cells in the non-adherent microenvironment of the bloodstream, known as circulating tumor cells (CTCs), has been detected in numerous epithelial cancers including breast, prostate, colon, and lung [1]. CTCs are an early indicator of clinical spread of disease and their levels correlate with decreased patient survival [2, 3]. Based on the increasing clinical relevance of CTCs, understanding their molecular profile is emerging as a new opportunity to gain insight on disease progression and patient prognosis beyond enumeration alone. Though progress has been made on technologies to enhance the identification and enumeration of CTCs [1, 4, 5], major limitations remain in performing downstream functional studies due to challenges with accurate detection and the low number of CTCs that can be retrieved from patient blood (frequency of approximately 1 in 100 million cells in the bloodstream) [1]. Some of the techniques currently being employed to analyze CTCs include fluorescence in situ hybridization, sequencing, immunostaining, xenograft transplantation, and RNA or protein-based analysis [1, 4, 6, 7]. However, these methods do not allow for real-time analysis of CTCs in an environment that preserves their free-floating nature.

Microscopy analysis of CTCs has focused almost exclusively on cells adhered to surfaces (glass, plastic, extracellular matrix (ECM)) owing to the ease of imaging and characterization of cells in these static positions. However, the functional and molecular characteristics of adherent and non-adherent tumor cells are dramatically different [8–11]. Thus a critical knowledge gap exists in the understanding of epithelial tumor cells in non-adherent microenvironments, such as those found in blood vessels. Non-adherent breast carcinoma cells, for example, produce unique tubulin-based microtentacles (McTNs) that promote tumor cell aggregation [12, 13], reattachment to endothelial layers [14, 15], and retention of CTCs in the lungs of mice [16, 17]. New enabling technologies to image tumor cells, McTNs, and other features in the absence of ECM attachment could vastly improve the understanding of dynamic cell behaviors that occur in the non-adherent microenvironments encountered by CTCs during metastasis. These tools could also support opportunities for selective targeting of drugs to McTNs or other structures presented preferentially by CTCs during metastatic spread, as well as help address rising concerns that chemotherapies meant to reduce tumor growth may actually increase metastatic risk [18]. Here, we exploited the discovery that McTNs form only when protein-based adhesions are absent to create an innovative platform for real-time imaging of the dynamic features of live, non-adherent tumor cells. This approach allows new types of information to be collected (e.g., McTN behavior on live cells over time) while reducing variables such as changes in cell function that occur during adhesion or the complexities of imaging cells in suspension that drift or are washed away during microfluidic flow.

Biomaterials offer many attractive features – stability, biocompatibility, versatile chemistries – for controlling cell adhesion. Common approaches include chemically functionalizing surfaces, incorporating cell adhesion peptides, and micropatterning using polymer-based soft lithography or electrospinning techniques. Of particular note, several recent studies have exploited biomaterials to identify CTCs [19–21] or used microfluidic devices to isolate and immobilize CTCs by acoustic separation, topography, controlled flow rates, and antibody traps [22–25]. Polyelectrolyte multilayers (PEMs) are nanoscale, polymeric materials assembled by electrostatic or hydrogen bonding interactions during a layer-by-layer (LbL) deposition process. PEMs can be coated on topographically-complex surfaces (e.g., colloidal, microfluidic) and offer programmable surface functionalities depending on the polymers used to assemble films. PEMs have recently been employed to capture CTCs through incorporation of cytophilic polymers or cell-adhesive proteins that promote CTCs adhesion [26–28]. However, new strategies are needed to study the dynamics of McTNs and other unique metastatic features that form only when CTCs are in non-adherent environments.

To enable this new ability, we identified three design features that would allow prolonged, real-time imaging and drug screening of McTNs on live tumor cells in a free-floating state: 1) optically-clear coatings to support imaging, 2) ability to control microfluidic flow over cells and 3) simple, low-energy manufacturing process. Past studies have demonstrated the utility of PEMs for tuning cell adhesion by varying polymer composition or through addition of lipids, RGD sequences, or other binding moieties [29–34]. Thus we leveraged PEMs to design a platform to immobilize live, detached tumor cells on microfluidic devices. We show that assembling cytophobic PEM films with cytophilic lipid tethers maintains the free-floating properties of tumor cells while providing spatial immobilization of cells. When tethered, McTNs on live cells can be visualized in real time and the dynamics of these structures can be assessed during microfluidic flow of drugs that enhance or destabilize McTNs. This technology could generate fundamental insight into a critical stage of metastasis that has been largely understudied due to technical challenges and support new approaches to exploit McTNs as biomarkers for the metastatic efficiency of tumor cells in diagnosis, prognosis, and targeted drug design.

## RESULTS

**Figure 1:**
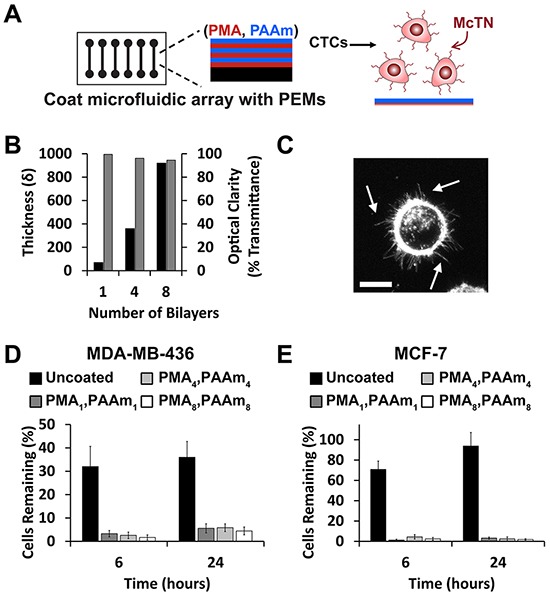
PEMs form a cytophobic layer allowing McTN visualization on microfluidic devices **A.** Schematic depicting coating of microfluidic slide with PEMs to maintain free-floating behavior of tumor cells. **B.** PEM coatings increase in thickness (left axis, black) with the number of bilayers but maintain optical clarity (right axis, gray). Data (mean ± SEM) correspond to samples in triplicate. **C.** Maximum intensity z-projection of MDA-MB-436 cells on PMA_4_/PAAm_4_ surfaces showing McTNs (arrows). Scale bar = 10μm. Percent of **D.** MDA-MB-436 and **E.** MCF-7 cells (mean ± SEM) remaining on surfaces after washing uncoated slides or slides coated with 1, 4, or 8 PMA/PAAm bilayers. Data represents mean values of three independent experiments.

The responses of cancer cells detached from ECM (i.e., in a circulating or free-floating stage) are highly important in survival, apoptosis, metastasis, and even in the expression of stem cell characteristics [36, 37]. However, tumor cells in this state are greatly understudied due to the technical and clinical challenges of continuously imaging cells not adhered to surfaces. Maintaining free-floating cell behavior of breast cancer cells is particularly critical in promoting McTN formation [12]. Thus we used breast tumor cells to first test if programming the compositions of PEM coatings would allow minimal tumor cell adhesion to maintain the free-floating characteristics (e.g., McTNs) of these cells (Figure 1A), before adding a lipid tether in subsequent designs.

### PEMs inhibit cell attachment allowing for McTN visualization

We formed PEMs from two common polymers, poly(methacrylic acid) (PMA) and polyacrylamide (PAAm) that have previously been shown to limit cell adhesion of numerous cell types [29, 38]. Substrates coated with PEMs offered precise control over film thickness and did not limit optical transmission, a feature important for pre-clinical and clinical imaging (Figure 1B). Since human breast tumor cells lines have not yet been tested on PEM-coated substrates, we first confirmed that PMA/PAAm multilayers could prevent cell adhesion in two NCI breast cancer cell lines, MDA-MB-436 and MCF-7. MDA-MB-436 cells seeded on slides coated with cytophobic PEMs maintained McTN display (Figure 1C), demonstrating for the first time maintenance of free-floating tumor cell behavior by using PEMs. We next coated multi-well culture plates or microfluidic slides with PEMs and allowed cells to attach for 0, 6, and 24 hours. The number of cells remaining after washing at each time point was then quantified by image analysis and cell proliferation (CellTiter). Imaging revealed that PEM-coatings prevented attachment of either cell line (Figure 1D, 1E, Supplementary Figure S1A, S1B) for at least 24 hours using 1, 4, and 8 PMA/PAAm bilayers. Cell proliferation data also indicated that deposition of 4 bilayers and 8 PMA/PAAm bilayers showed reduced attachment compared with 1 bilayer for both lines (Supplementary Figure S2A, S2B). Four bilayer films were prioritized for future experiments since these films formed cytophobic surfaces that most efficiently decreased cell attachment while maintaining McTN activity. Coatings did not impact the viability of either cell line, regardless of substrate (Supplementary Figures S2C–S2F, S3, S4).

### Modification of PEMs with lipid tethers retains tumor cells after washing

**Figure 2:**
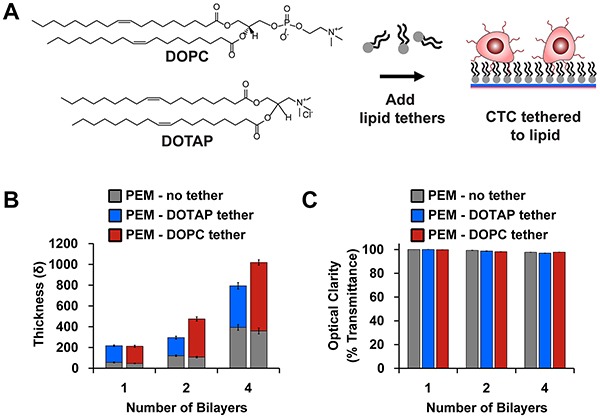
Modification of PEMs with lipid tethers **A.** Schematic depicting how lipid-terminated PEMs promote interaction with tumor cell membranes. **B.** Film thickness and **C.** optical clarity (mean ± SEM) after addition of DOTAP (blue) and DOPC [red]. Lipids promote growth of film while maintaining an optically-clear substrate for imaging. Data correspond to the mean of samples prepared in triplicate with three measurements per surface.

**Figure 3:**
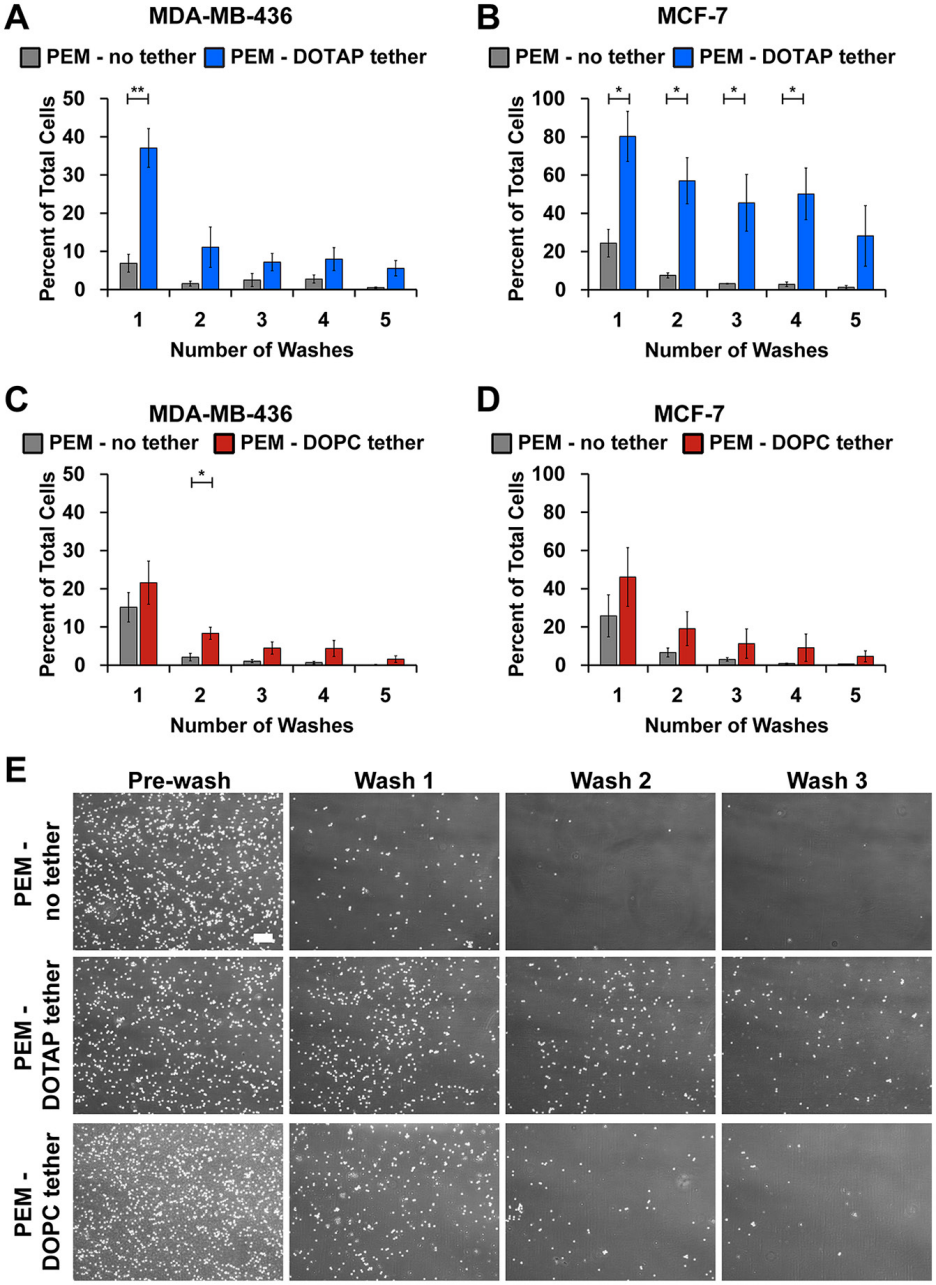
DOTAP tethers breast cancer cells Percent cell retention of **A.** MDA-MB-436 and **B.** MCF-7 cells plated on microfluidic slides coated with PMA_4_/PAAm_4_ bilayers alone or with DOTAP. Percent cell retention of **C.** MDA-MB-436 and **D.** MCF-7 cells plated on microfluidic slides coated with PMA_4_/PAAm_4_ bilayers alone or with DOPC. The remaining cells after each wash was quantified with CellProfiler and normalized to the initial cell number. Data represents mean of triplicate independent experiments (mean ± SEM). **E.** Representative images of MDA-MB-436 cells at time 0 and after 3 subsequent washes on PMA_4_/PAAm_4_ bilayers with no tether and with PEM-DOTAP or PEM-DOPC tethers at 4x magnification. Scale bar = 200μm. *P<0.05 **P<0.01.

Although PEM-coated surfaces prevented tumor cell attachment and supported free floating behavior, these cells were removed during washing with buffer. Thus we sought to develop a strategy to maintain McTNs while also providing spatial localization during microfluidic flow for real-time imaging and drug screening. We hypothesized that the addition of a terminal lipid layer that interacts with cell membranes would loosely tether cells to the surface during microfluidic flow. We tested 1,2-dioleoyl-3-trimethylammonium-propane (DOTAP) and 1,2-dioleoyl-*sn*-glycero-3-phosphocholine (DOPC) as cationic and zwitterionic lipids, respectively, owing to the ability of these molecules to interact with the PEMs electrostatically (Figure 2A). Following addition of DOTAP or DOPC, total film thickness increased, though individual bilayers were still only 20nm and optical clarity remained high (Figure 2B, 2C). We next tested if the addition of lipid supported tethering of breast tumor cells without inhibiting free-floating features such as McTNs. Tumor cells were seeded on microfluidic slides coated with (PMA/PAAm)_4_ without lipids (PEM-no tether), or with either terminal lipid layer (PEM-DOPC tether, PEM-DOTAP tether). During successive wash steps, DOTAP retained tumor cells more efficiently compared to non-tethered cells seeded on microfluidic slides coated with PEM only (Figure 3A, 3B). MCF-7 cells exhibited significantly higher overall cell retention with PEM-DOTAP compared to PEM only over four washes. MDA-MB-436 cells showed significant retention of cells with PEM-DOTAP for the first wash and continued to tether around 10% of cells for subsequent washes. Tethering was dependent on lipid composition, as after five washes, DOTAP tethered and retained 30% of MCF-7 cells, while DOPC was ineffective at tethering cells during successive washing (Figure 3A, 3B versus Figure 3C, 3D). Representative images of cells from each line tethered on these surfaces are shown in Figure 3E and Supplementary Figure S5. Since DOTAP demonstrated superior tethering for both types of tumor cells, this lipid was prioritized for functional assays.

### Lipid tethers preserve McTNs and cell viability

**Figure 4:**
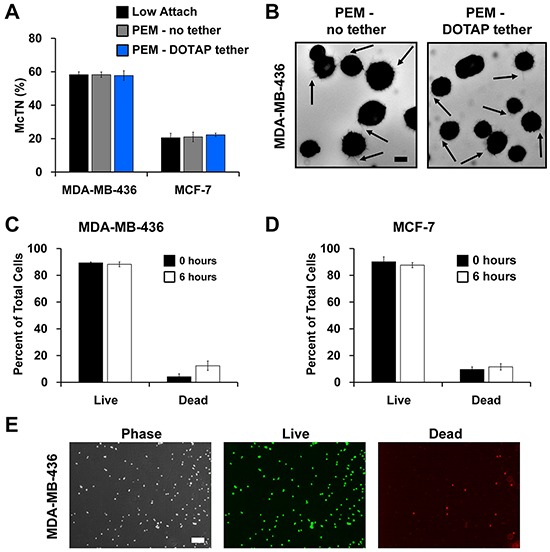
Lipid tethering retains free-floating characteristics of breast tumor cells and does not affect cell viability **A.** McTN quantification of MDA-MB-436 and MCF-7 cells suspended on a low-attach plate, microfluidic slides with PEM-no tether, and microfluidic slides with PEM-DOTAP tether. Data represents blinded quantification of McTN frequency from three independent experiments with 100 cells counted for each (mean ± SEM). **B.** Representative images of McTNs (arrows) on MDA-MB-436 cells seeded on PEM-no tether and PEM-DOTAP tether microfluidic slides at 40x magnification. Scale bar = 10μm. Viability of **C.** MDA-MB-436 and **D.** MCF-7 cells calculated at 0 and 6 hrs after seeding on microfluidic slides with PEM-DOTAP tether. Data represents mean cell viability from three independent experiments (mean ± SEM). **E.** Representative images show viability of MDA-MB-436 cells tethered by DOTAP for 6 hrs. Phase contrast, live (green fluorescence), and dead (red fluorescence) images taken at 4x magnification. Scale bar = 200μm.

We next determined if lipid tethering with DOTAP maintained free-floating tumor cell characteristics. As a first indicator, McTN frequency was assessed on PEM and PEM-DOTAP surfaces. Blinded McTN counts revealed no differences in McTN frequency on PEM-no tether and PEM-DOTAP surfaces compared to previously published counts on low-attach multi-well plates (Figure 4A). These results indicate lipid tethering does not impact the ability of MDA-MB-436 cells to assemble McTNs, results confirmed by epifluorescence imaging of McTNs on cells incubated with each type of PEM or substrate (Figure 4B, Supplementary Figure S6A). MCF-7 cells exhibited similar results (Figure 4A, Supplementary Figure S6B), though these cells assembled McTN at lower frequencies, as previously reported [39]. Further, toxicity studies confirmed tethering does not impact tumor cell viability (Figure 4C–4E, Supplementary Figure S6C–S6F). Thus for the first time, our approach allows maintenance of free-floating tumor cell behavior while spatially fixing the location of tumor cells.

### Lipid tethers allow for real-time high resolution McTN imaging in response to drug treatments

**Figure 5:**
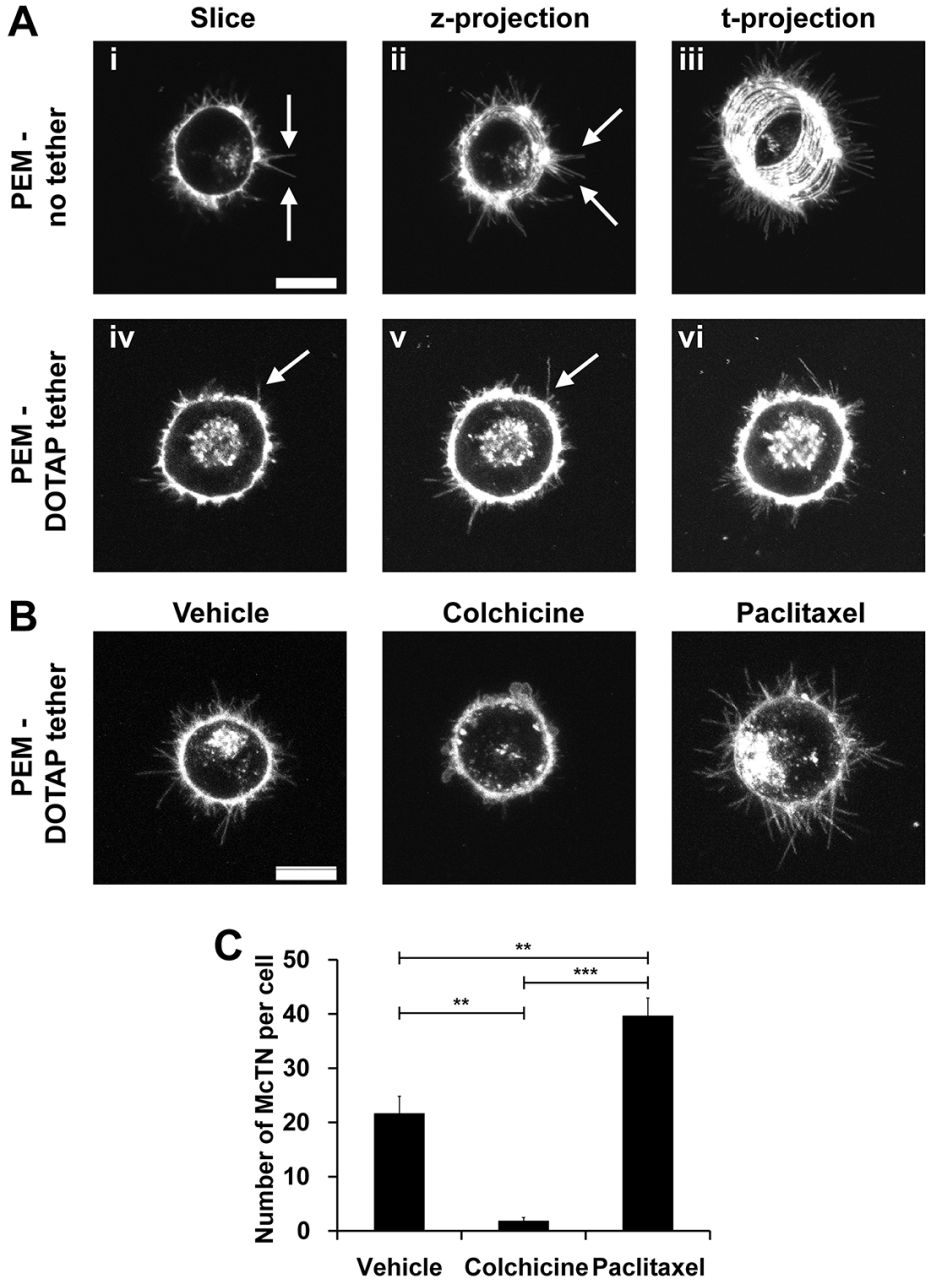
Lipid tethering allows for real-time McTN imaging in response to drug treatment and minimizes effects of drift **A.** McTN (arrows) imaging of MDA-MB-436 cells seeded on microfluidic slides with PEM-no tether (i-iii) and PEM-DOTAP tether (iv-vi). Representative 1μm slice (i and iv), maximum z-projection of 5 slices at one time point (ii and v), and maximum t-projection after 20 frames (iii and vi) are shown at 60x magnification. **B.** McTN (arrow) imaging of MDA-MB-436 cells seeded on microfluidic slides with PEM-DOTAP tether after treatment with 5μM colchicine for 15 mins and 1μg/ml paclitaxel for 120 mins. Maximum intensity z-projections of five 1μm slices at one time point are shown at 60x magnification. Complete time-lapse movies are available in Fig. S7. Scale bar = 10μM. **C.** Manual quantification of the average number of McTNs per cell (mean ± SEM) in MDA-MB-436 cells treated with colchicine and paclitaxel. **P<0.01 ***P<0.001.

One of the greatest challenges in studying free-floating cells is the difficulty in measuring their functional properties or behavior in real time. This is especially apparent when trying to image free-floating cells over time and in three dimensions. Epifluorescence is unable to capture McTNs in high resolution (Figure 4B). Confocal microscopy of cells labeled with a fluorescent membrane dye improves signal to noise and allows McTNs to be imaged with high contrast (Figure 5Ai, arrows). However, since McTNs occur on free-floating cells, the time required to generate a 3-dimensional stack of z-slice images for tracing McTN length yields significant blurring as free-floating cells drift over a surface to which they cannot attach. This limitation was encountered when imaging cells exposed to PEM surfaces without lipid tethers (Figure 5Aii, arrows). The blurring effect of cell drift is even more apparent across a time projection (Figure 5Aiii). In contrast, tethered breast tumor cells not only maintained McTNs (Figure 5Aiv, arrow), but also eliminated blurring of McTNs in z-stacks. This strategy also allowed microtentacle length to be traced efficiently across z-stacks (Figure 5Av, arrow) and limited drift of the cell body during time-lapse imaging (Figure 5Avi). These phenomena are evident during time-lapse movies of drifting tumor cells seeded on PEM-no tether surfaces, whereas DOTAP tethering caused cells to remain fixed in one location while preserving McTN dynamics (Supplementary Figure S7A, S7B). It is interesting to note that debris is seen moving quickly through the field throughout the movie while the cell remains immobile and centered (Supplementary Figure S7B).

The major advantage in imaging McTNs over time is being able to study their responses to drugs not only by McTN frequency, but also McTN dynamics. To demonstrate this potential, we recorded three dimensional z-stacks of untreated MDA-MB-436 cells, cells treated with the microtubule destabilizing agent, colchicine, or the microtubule stabilizing agent, paclitaxel. Addition of colchicine decreased McTNs while paclitaxel enhanced McTNs (Figure 5B). Over time, colchicine shrunk McTNs and increased cell blebbing, whereas paclitaxel hyperstabilized McTNs, dramatically decreasing their dynamics compared to vehicle control. These new trends are clearly observable in high resolution movies (Supplementary Figure S7C–S7E) that would be otherwise impossible without lipid tethering, since microfluidic flow would wash these cells away or drift would cause blurring. This strategy also creates new opportunities to study free-floating cell properties on a per-cell basis. For example, we measured time-dependent drug response and discovered that treatment with colchicine decreased the mean number of McTN per cell from 21.6 ± 7.2 to 1.8 ± 1.5, while paclitaxel treatment increased the frequency of these structures to 39.6 ± 7.5 McTNs/cell (Figure 5C).

## DISCUSSION

The majority of cancer-related deaths are due to the spread of tumor cells through the circulation from the primary site to a secondary organ [40]. While in the circulation, tumor cells are in a non-adherent microenvironment that is unlike the conditions in a primary tumor or the metastatic site. In these non-adherent conditions, tumor cells undergo many biochemical and structural changes that affect their sensitivity to therapies and their overall metastatic efficiency [15, 41]. Classical drug studies and microscopy focus on analyzing tumor cells attached to a substrate due to the practical ease of analyzing cells under static conditions, but these methods do not recapitulate the free-floating environment of CTCs. Therefore, we have developed a microfluidic device that can anchor tumor cells using a lipid moiety while preventing their attachment to a substrate.

In this proof-of-concept study we show that tethering is an effective way to retain the free-floating behavior of cancer cells and provides new opportunities to study their functional properties with high-resolution microscopy through spatial localization. This strategy offers the ability to coat a variety of surfaces, including microfluidics. We show that incorporating a lipid moiety on PEMs can passively immobilize tumor cells in a manner that preserves McTN formation and does not affect cell viability. In this method, no cellular adhesive properties are necessary because the interaction of the cell membrane with the lipid results in cell tethering. Lipids have previously been used to immobilize cells [32, 34]; here we have advanced this idea to create a simple system for studying McTNs on free-floating tumor cells. This approach eliminates the need for solvents, patterning, designed topographies, or antibodies used in other recent studies aimed at promoting adhesion of CTCs. Further, cells can be tethered in complete media and in a short time frame, both enabling real-time study of McTNs on individual CTCs. Therefore, using this technique, tethering of any cell type can be achieved in a simple and rapid manner, making this a robust platform to study free-floating behaviors across various cell types.

In our studies, each PEM lipid formulation tethered cancer cells, but the lipid moiety itself altered cell retention, with DOTAP – a cationic lipid – driving the best tethering. DOPC – a zwitterionic lipid – contains a phosphate group which may cause less favorable interactions with cell membranes compared to DOTAP. While cells were immobilized on the substrate, they remained free-floating owing to the cytophobic nature of the initial PEM coating. There was some loss of tethered cells as a function of the number of washes, but it is likely these interactions can be strengthened by incorporation of cross-linkable lipids [32, 34, 42, 43]. We also observed a difference in the tethering efficiency as a function of cell line, with MCF-7 breast cancer cells exhibiting better tethering compared to MDA-MB-436 cells. These changes may be due to the differences in membrane composition or surface marker expression of each cell line [44].

We demonstrate the utility of tethering for tumor cell analysis by assessing the response of McTNs to microtubule-targeting drugs. Unlike our previous studies, it is now possible to add drugs via microfluidic exchange without displacing cells, allowing measurements of colchicine-dependent McTN disruption [12] and paclitaxel-dependent McTN enhancement [18] to be measured with time-lapse imaging for the first time. Our discovery that paclitaxel hyperstabilizes McTNs, for example, is significant since microtubule stability can enhance McTN formation and increase the re-attachment efficiency of tumor cells [18]. Mouse models of metastasis indicate that this increased McTN formation and tubulin stability results in greater lung trapping of tumor cells [12, 16, 18]. Therefore, analyzing McTN dynamics and their response to drugs has important implications on the metastatic ability of tumor cells. These technological advances should allow many additional quantitative McTN metrics to be accurately measured (length, dynamics, etc.) and also improve on qualitative observations that have until now not been possible on a live, individual cell basis.

High numbers of CTCs correlate with increased metastasis and decreased survival of patients with metastatic cancer [2, 3, 45, 46]. However, CTC enumeration alone may not be a good marker for disease staging and prognosis [45]. Therefore, improved biologic characterization of CTCs is necessary to better understand their clinical value. Numerous new approaches have been designed to improve CTC detection and enumeration, but the ability to study the functional properties of CTCs remains difficult [1]. *Ex vivo* culture of CTCs in non-adherent conditions has provided one method to analyze CTCs from patients [6]. This PEM-lipid tethering technology may be applied to these culturing methods to keep cells from adhering, but offers the unique capabilities of rapid single-cell analysis through staining and imaging in real-time.

Studying the biology of CTCs has suggested important consequences for both metastatic efficiency and the sensitivity of these structures to candidate cancer drugs. Of note, patterns of drug sensitivities have been linked to the genetic mutations present in individual CTC samples from breast cancer and lung cancer patients, indicating that a change in tumor genotypes during the course of treatment can lead to drug resistance [6, 41, 47]. Our work shows tethering tumor cells allows rapid analysis of specific drug responses in real-time. Markers of epithelial-to-mesenchymal transition (EMT) are also upregulated in CTCs with mesenchymal markers specifically enriched in CTC clusters. These clusters have increased metastatic capabilities compared with single cells alone [7, 48]. Thus our approach can be applied to these existing techniques for fundamental CTC studies at the single-cell level. Assessing the effects of drugs on cell viability, EMT markers, or McTNs could all have implications on their metastatic phenotype. Tethering would also allow these studies to be conducted in a manner that more closely recapitulates the free-floating environment found in circulation. Though our study focuses on the analysis of tumor cells, this simple and rapid tethering technology is translatable to numerous other cell types that are encountered in the blood stream (e.g., red blood cells, platelets, lymphocytes, macrophages) and may function differently in a free-floating environment. With new technologies, CTCs will play an increasing role in informing therapy and disease progression of cancer patients. Toward this goal, tethering CTCs with PEM-lipid films could serve as a new tool to analyze CTC samples to provide better personalized treatment decisions for patients.

## MATERIALS AND METHODS

### Cell lines & materials

MDA-MB-436 and MCF-7 cell lines were purchased from ATCC and cultured with Dulbecco's Modified Eagle Medium supplemented with 10% fetal bovine serum and 1% penicillin-streptomycin solution. Poly(methacrylic acid) (MW 100,000) and polyacrylamide (PAAm) (MW 5,000,000-6,000,000) were purchased from Polysciences. Poly(allylamine hydrochloride) (PAH) (MW ∼200,000) was purchased from Alfa Aesar. 1,2-dioleoyl-3-trimethylammonium-propane (chloride salt) (DOTAP) and 1,2-dioleoyl-*sn*-glycero-3-phosphocholine (DOPC) were purchased from Avanti Polar Lipids. Colchicine was purchased from Sigma and paclitaxel was purchased from Enzo Life Sciences.

### PEM film deposition and characterization on planar substrates

For multilayer film deposition, similar to methods previously reported [35], PMA and PAAm were prepared as 0.01M solutions using ultrapure water and adjusted to pH 3. All polymer solutions were filtered with a 0.45 μm cellulose nitrate filter prior to use in multilayer film assembly. For planar substrates, quartz (Chemglass Life Sciences) or silicon (Silicon Inc.) were cut into 5mm × 25mm substrates using a dicing saw (Model 1006, Micro Automation). Cut substrates were cleaned with sequential washing with acetone, ethanol, methanol, and deionized water then charged using an oxygen plasma Jupiter III system (March). These substrates were first immersed in the polycationic solution PAH (0.05M) for 15 mins then rinsed twice using two separate baths of deionized water at pH 3 to remove any excess polymer. This primer layer was followed by immersion of the substrates into polyanionic PMA (0.01M) for 5 mins followed by rinsing as above. The substrates were then immersed in a polycationic solution of PAAm (0.01M) for 5 mins and rinsed. For additional bilayers, the process was repeated without the addition of the primer layer (PAH) until the desired number of bilayers was assembled. Lipid formulations comprised of 1,2-dioleoyl-sn-glycero-3-phosphocholine (DOPC) or 1,2-dioleoyl-3-trimethylammonium-propane (DOTAP) were obtained from Avanti Polar Lipids. These lipids were prepared as 0.01M solutions with pH 3 deionized water and sonicated for 60 mins in a room temperature water bath. PEMs with a lipid tether were prepared by immersing PEM coated substrates in each lipid solution for 5 mins followed by two rinsing steps. The final, coated substrates were removed from solution, blown dry with compressed, filtered air, and stored at room temperature prior to characterization. Film thickness and optical clarity after deposition onto silicon and quartz substrates were measured using a LSE stokes ellipsometer (Gaertner Scientific Corportation) and by measuring light transmittance at 600nm using an Evolution 60 UV-visible spectrophotometer (Thermo Scientific), respectively.

### PEM film deposition on microfluidic slides and multi-well plates

Uncoated microfluidic slides (1μ-Slide VI 0.4) were obtained from Ibidi and tissue culture treated 96-well plates were obtained from Corning. To coat the microfluidic slides, 120μL of each polyelectrolyte solution was added to the microchannels and 75μl of solution was added to each well of the multi-well plate. After incubation, solution was removed via aspiration and rinsed twice for 1 min using 120μL of pH 3 water. Bilayers of PMA and PAAm were assembled and terminated with either DOPC or DOTAP as described above. Following deposition, slides were allowed to air dry for 1 hr at room temperature then stored at room temperature.

### Attachment image analysis

MDA-MB-436 and MCF-7 breast cancer cells were seeded on PEM coated microfluidic slides (50,000 cells/channel) ranging from 0 to 8 bilayers. An Olympus CKX4 microscope was used for all experiments to capture images at 4x magnification. Three pictures per channel were taken after cell seeding for each condition to quantify initial cell number (t0). At 6 and 24 hrs, media was removed from the channel and the channel was washed once before addition of new media. Three images per channel were taken for each condition. The area of the image occupied with cells (as a percent) was quantified using CellProfiler (Broad Institute) and the average from three images was calculated. The average percentage for each condition was then normalized to the area occupied at t0.

### Attachment cell titer

MDA-MB-436 and MCF-7 breast cancer cells were seeded on PEM coated 96-well plates (20,000 cells/well) ranging from 0 to 8 bilayers. At each time point (1, 3, 6, and 24 hrs), media was removed from the well and the well was washed once before addition of new media. After the 24hr time point an additional wash was done on all wells. Cell number was determined using CellTiter reagent according to manufacturer's instructions. Each time point was normalized to initial cell number from a reading done immediately after cell seeding.

### PEM viability

MDA-MB-436 and MCF-7 breast cancer cells were seeded on PEM coated microfluidic slides with PMA_4_/PAAm_4_ bilayers (50,000 cells/channel). At 0, 6, and 24 hrs Live/Dead (Life Technologies) reagent was added according to manufacturer's instructions. Corresponding phase contrast, live (calcein-AM) green fluorescence, and dead (ethidium homodimer-1) red fluorescence images were taken in triplicate at 4x magnification with an Olympus CKX41 fluorescence microscope. The number of cells in each image was quantified using CellProfiler. The percent of live and dead cells were calculated by quantifying the number of green fluorescence positive and red fluorescence positive cells, respectively, and dividing by total number of cells in the phase contrast image. GFP and Texas Red filters were used to for imaging. MDA-MB-436 and MCF-7 breast cancer cells were plated on 96-well black plates with PMA_4_/PAAm_4_ bilayers (20,000 cells/well). At time 0, 1, 3, 6, and 24 hours Live/Dead reagent was added and read on a plate reader according to manufacturer's instructions. Relative fluorescence units (RFU) were normalized to time 0.

### Tethering washing

MDA-MB-436 and MCF-7 cells were seeded on PMA_4_/PAAm_4_ coated microfluidic slides with DOPC or DOTAP (50,000 cells/channel). Cells were incubated for 1 hr to allow for tethering. To quantify initial cell number, three images per channel were taken for each condition at time 0. After 1 hr, existing media was gently removed from the bottom port of each channel and fresh media was added to the top port. Following a wash, three images were taken per channel for each condition using an Olympus CKX41 microscope at 4x magnification. This process was repeated for each wash. The area of the image occupied with cells (as a percent) was quantified using CellProfiler and the average from three images was calculated. The average percentage for each condition was then normalized to the area occupied at time 0.

### Tethering viability

MDA-MB-436 and MCF-7 cells were seeded on PMA_4_/PAAm_4_ coated microfluidic slides with DOPC or DOTAP (50,000 cells/channel). Cells were incubated for 1 hr to allow for tethering. After 1 hr, one wash was done where the existing media was gently removed from the bottom port of each channel and fresh media was added to the top port. This wash was to ensure only tethered cells were analyzed. At 0 and 6 hrs after washing, Live/Dead reagent was added according to manufacturer's instructions. Corresponding phase contrast, live (calcein-AM) green fluorescence, and dead (ethidium homodimer-1) red fluorescence images were taken in triplicate. The number of cells in each image was quantified using CellProfiler. The percent of live and dead cells were calculated by quantifying the number of green fluorescence positive and red fluorescence positive cells, respectively, and dividing by total number of cells in the phase contrast image. GFP and Texas Red filters were used for imaging.

### McTN counting

MDA-MB-436 cells were trypsinized, spun down, and resuspended in phenol red-free and serum-free DMEM. Cells were seeded on PMA_4_/PAAm_4_ coated microfluidic slides, PMA_4_/PAAm_4_ coated microfluidic slides with DOTAP, or a low attach 24-well plate (50,000 cells/channel). Cells were incubated for 1 hr to allow for tethering. After 1 hr, one wash was done where the existing media was gently removed from the bottom port of each channel and fresh media was added to the top port on the DOTAP slides. This wash was to ensure only tethered cells were analyzed. After this wash, CellMask Orange (Life Technologies) cell membrane dye was added to each channel to a final concentration of 1:10,000. McTNs were scored blindly in a population of 100 cells/well as previously described [12]. Representative images were taken at 40x magnification with an Olympus CKX41 fluorescence microscope.

### Imaging drift and drug treatments

MDA-MB-436 cells were trypsinized, spun down, and resuspended in phenol red-free and serum-free DMEM. Cells were seeded on PMA_4_/PAAm_4_ coated microfluidic slides and PMA_4_/PAAm_4_ coated microfluidic slides with DOTAP (50,000 cells/channel). Cells were incubated for 1 hr to allow for tethering. After 1 hr, one wash was done where the existing media was gently removed from the bottom port of each channel and fresh media was added to top port on the DOTAP slides. This wash was to ensure only tethered cells were analyzed. After this wash, CellMask orange cell membrane dye was added to each channel to a final concentration of 1:10,000. Cells were treated with 5μM colchicine for 15 mins and 1μg/ml paclitaxel for 120 mins. McTN imaging was done on an Olympus FV100 confocal laser scanning microscope at 60x magnification. Five 1μm slices and 20 frames at a 10 sec frame rate were taken for at least five image sets for each condition. The number of McTNs on each cell was manually counted on five cells per condition using the maximum intensity z-projection at the last frame.

### Statistical analysis

Graphpad Prism (version 6.02) was used to determine all statistic comparisons. Student's t-test and one-way ANOVA tests were performed with a Tukey multiple comparisons post-test as indicated. A p-value of 0.05 or less was considered statistically significant.

## SUPPLEMENTARY FIGURES AND FILE




