# Supplementary material for: Lipid tethering of breast tumor cells enables real-time imaging of free-floating cell dynamics and drug response
Source: Oncotarget. 2016 Feb 8;7(9):10486–97. doi: 10.18632/oncotarget.7251 (PMC4891134; doi:10.18632/oncotarget.7251)
Supplement: Supplementary file 1 [file oncotarget-07-10486-s001.pdf]

SUPPLEMENTARY FIGURES

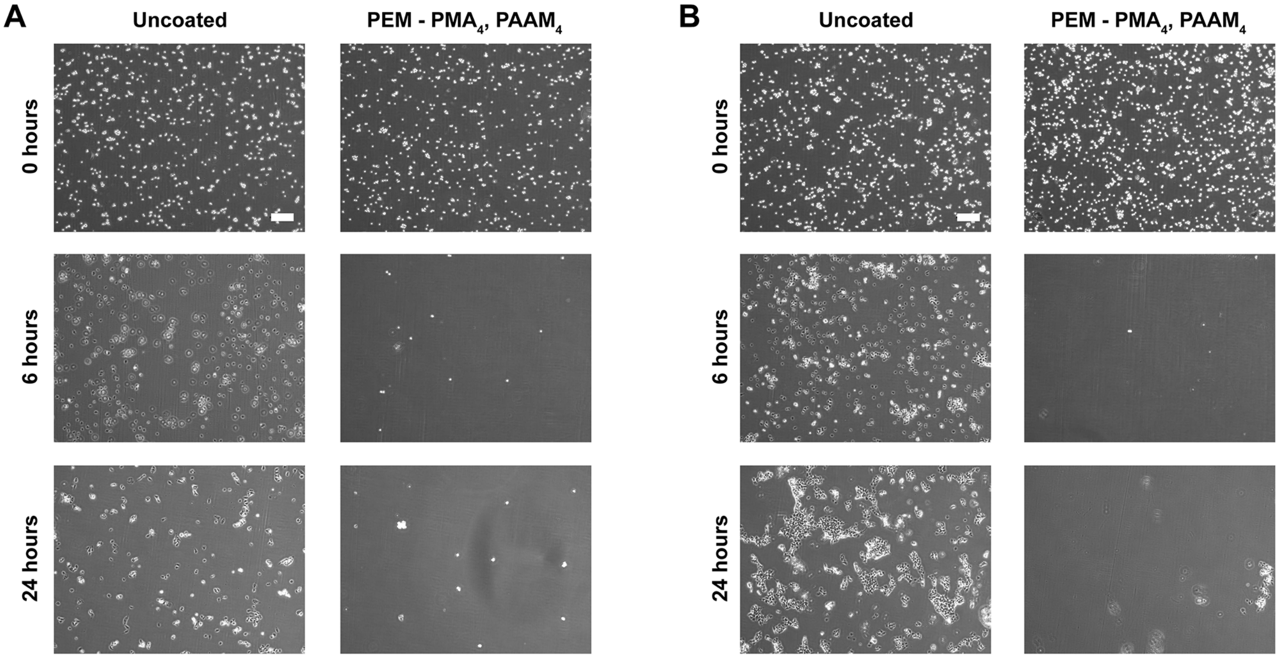

Supplementary Figure S1: PEM prevents attachment of MDA-MB-436 and MCF-7 breast cancer cells. Representative images of **A.** MDA-MB-436 and **B.** MCF-7 cells on microfluidic slides with 0 (uncoated) and PMA_4/$PAAm_4$ bilayers after one wash at 0, 6, and 24 hrs at 4x magnification. Scale bar represents 200μm.

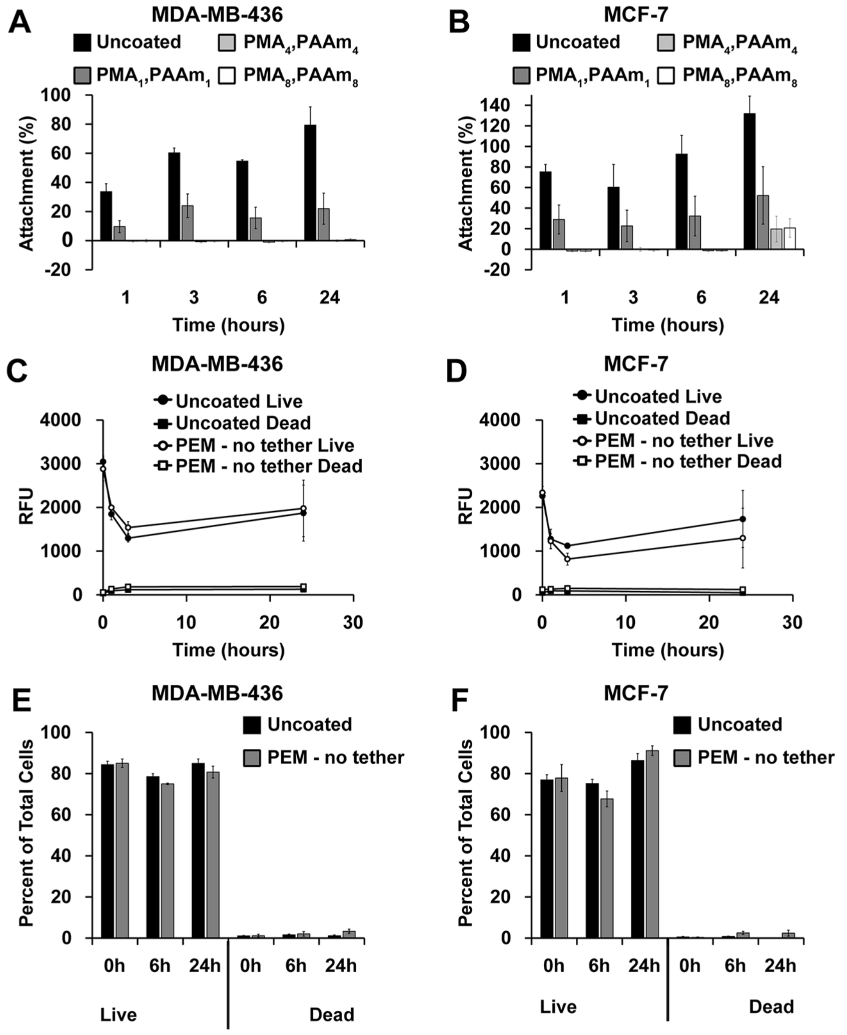

Supplementary Figure S2: PEM prevents cell attachment and does not affect cell viability. CellTiter analysis of the number of **A.** MDA-MB-436 and **B.** MCF-7 cells remaining on 96-well plate coated with 0 (uncoat), 1, 4, and 8 PMA/PAAm bilayers after one wash at 1, 3, 6, and 24 hrs normalized to initial cell number. Data represents mean cell attachment from three independent experiments (mean ± SEM). Viability of **C.** MDA-MB-436 and **D.** MCF-7 cells on a 96-well plate coated with 0 (uncoated), 1, 4, and 8 PMA/PAAm bilayers at 0, 1, 3, 6, and 24 hrs. Data represents mean viability from three independent experiments (mean ± SEM). Viability of **E.** MDA-MB-436 and **F.** MCF-7 cells plated on microfluidic slides with 0 (uncoated) or $PMA_4/PAAm_4$ bilayers calculated at 0, 6, and 24 hrs. Green fluorescence (live) and red fluorescence (dead) positive cells were quantified for each and divided by total cell number to calculate percent of live and dead cells, respectively using CellProfiler. Data represents mean viability from three independent experiments (mean ± SEM).

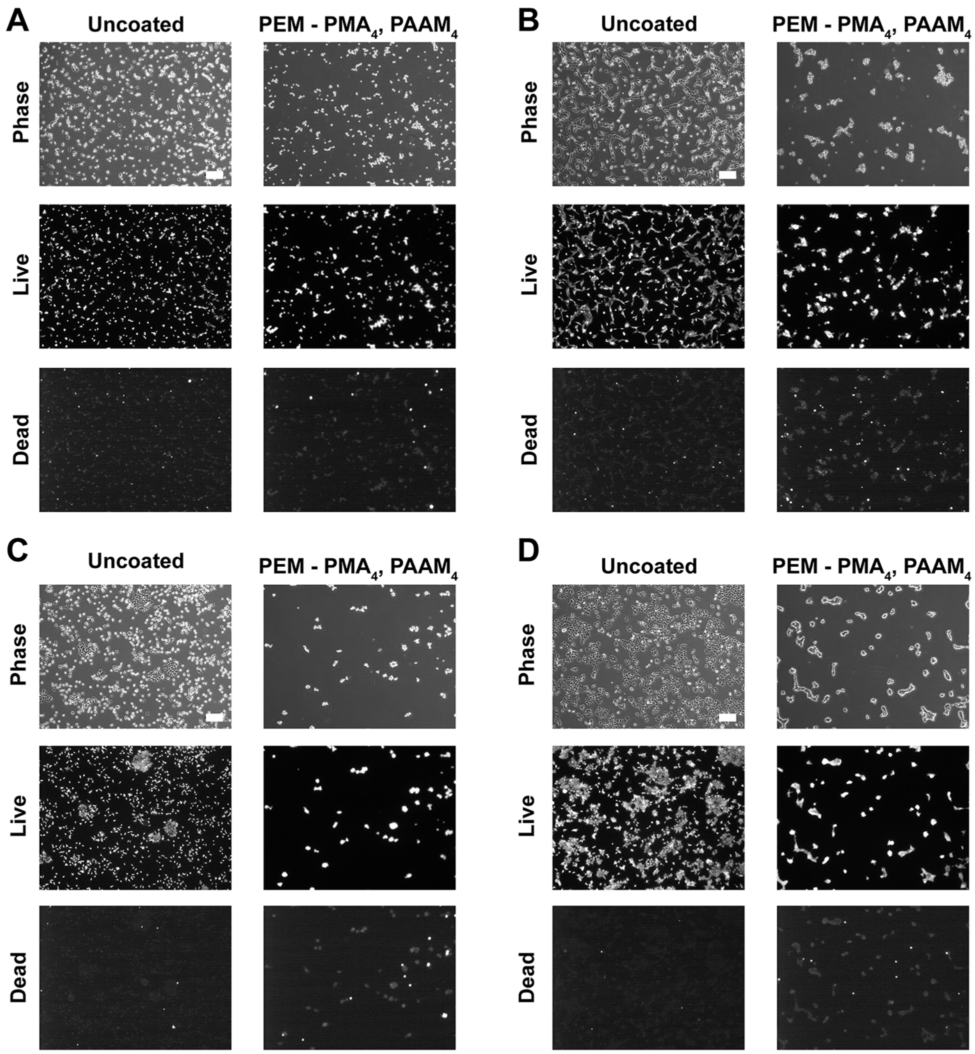

Supplementary Figure S3: PEM does not affect viability of MDA-MB-436 and MCF-7 cells. Representative images of the viability of MDA-MB-436 plated on microfluidic slides with 0 (uncoated) or $PMA_4/PAAm_4$ bilayers at **A.** 6 hrs and **B.** 24 hrs. Representative images of the viability of MCF-7 plated on microfluidic slides with 0 (uncoated) or $PMA_4/PAAm_4$ bilayers at **C.** 6 hrs and **D.** 24 hrs. Phase contrast, live (green fluorescence), and dead (red fluorescence) images taken at 4x magnification. Scare bar represents 200µm.

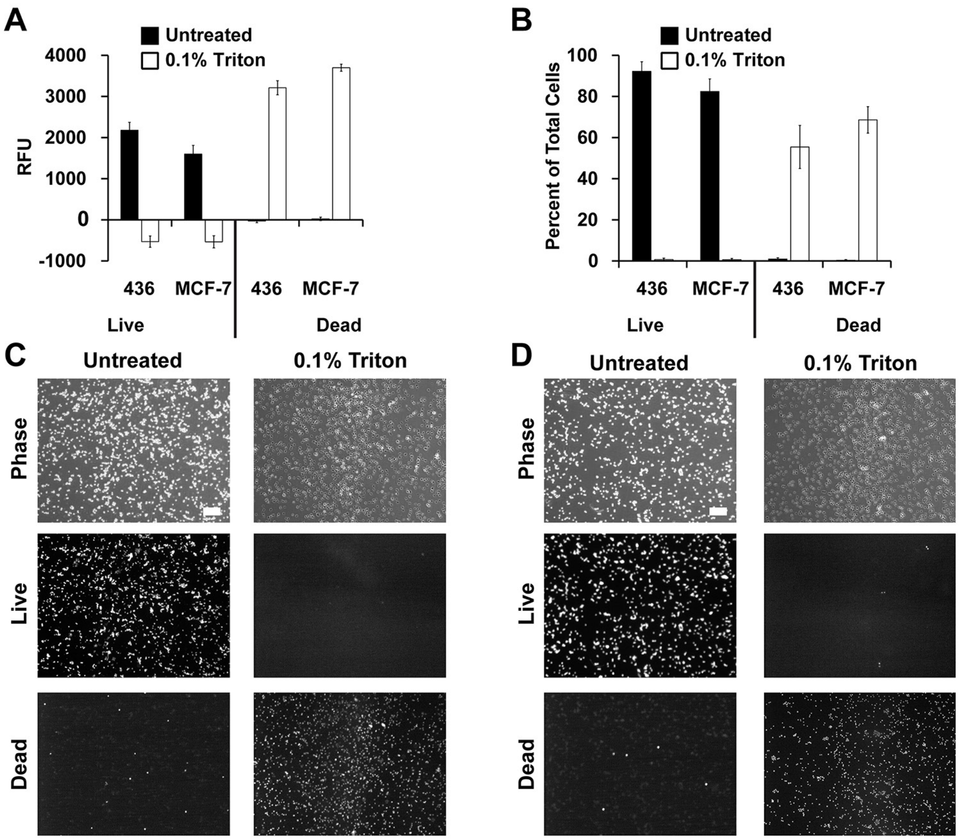

Supplementary Figure S4: Triton-X is a positive control for cell death. Cell viability of MDA-MB-436 and MCF-7 cells treated with 0.1% Triton-X plated on **A.** 96-well plate and **B.** microfluidic slides. Green fluorescence (live) and red fluorescence (dead) positive cells were quantified for each and divided by total cell number to calculate percent of live and dead cells, respectively, using CellProfiler. Data represents mean viability from three independent experiments (mean ± SEM). Representative images of **C.** MDA-MB-436 and **D.** MCF-7 cells treated with 0.1% Triton-X. Phase contrast, live (green fluorescence), and dead (red fluorescence) images taken at 4x magnification. Scale bar represents 200µm.

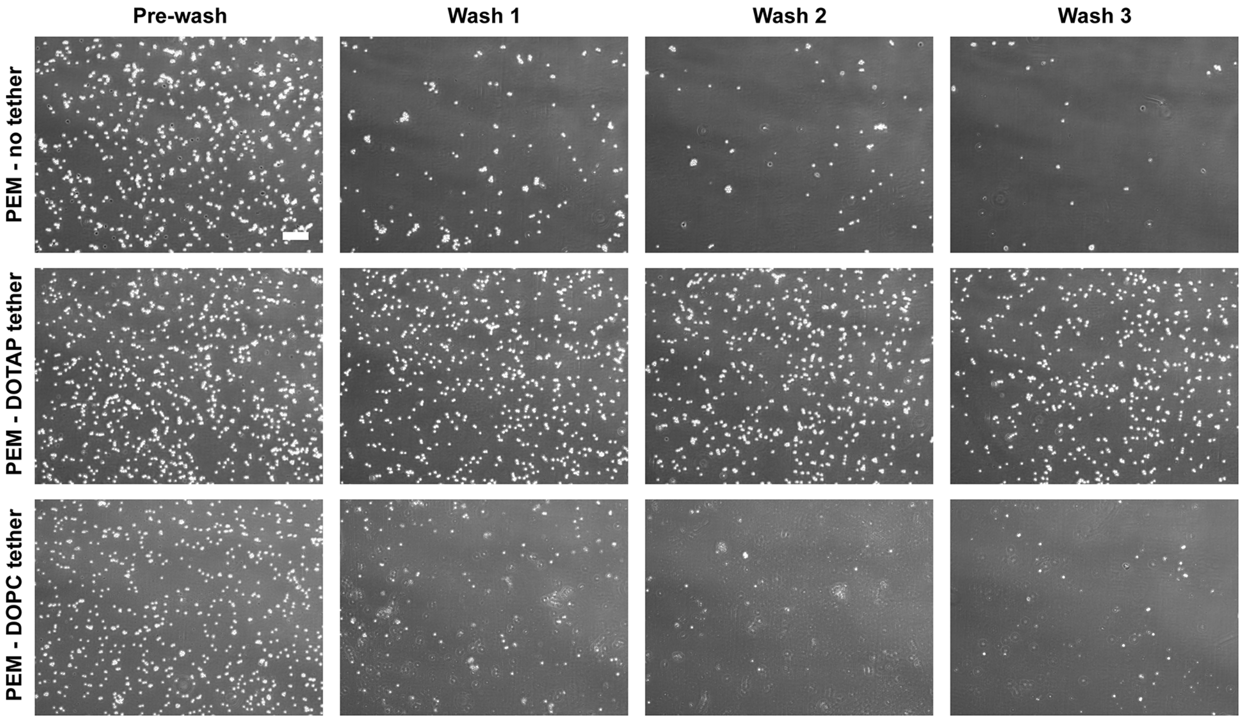

Supplementary Figure S5: DOTAP can tether MCF-7 breast tumor cells. Representative images of MCF-7 cells seeded on microfluidic slides with PEM-no tether, PEM-DOTAP tether, and PEM-DOPC tether prior to washing and after 3 subsequent washes at 4x magnification. Scale bar represents 200µm.

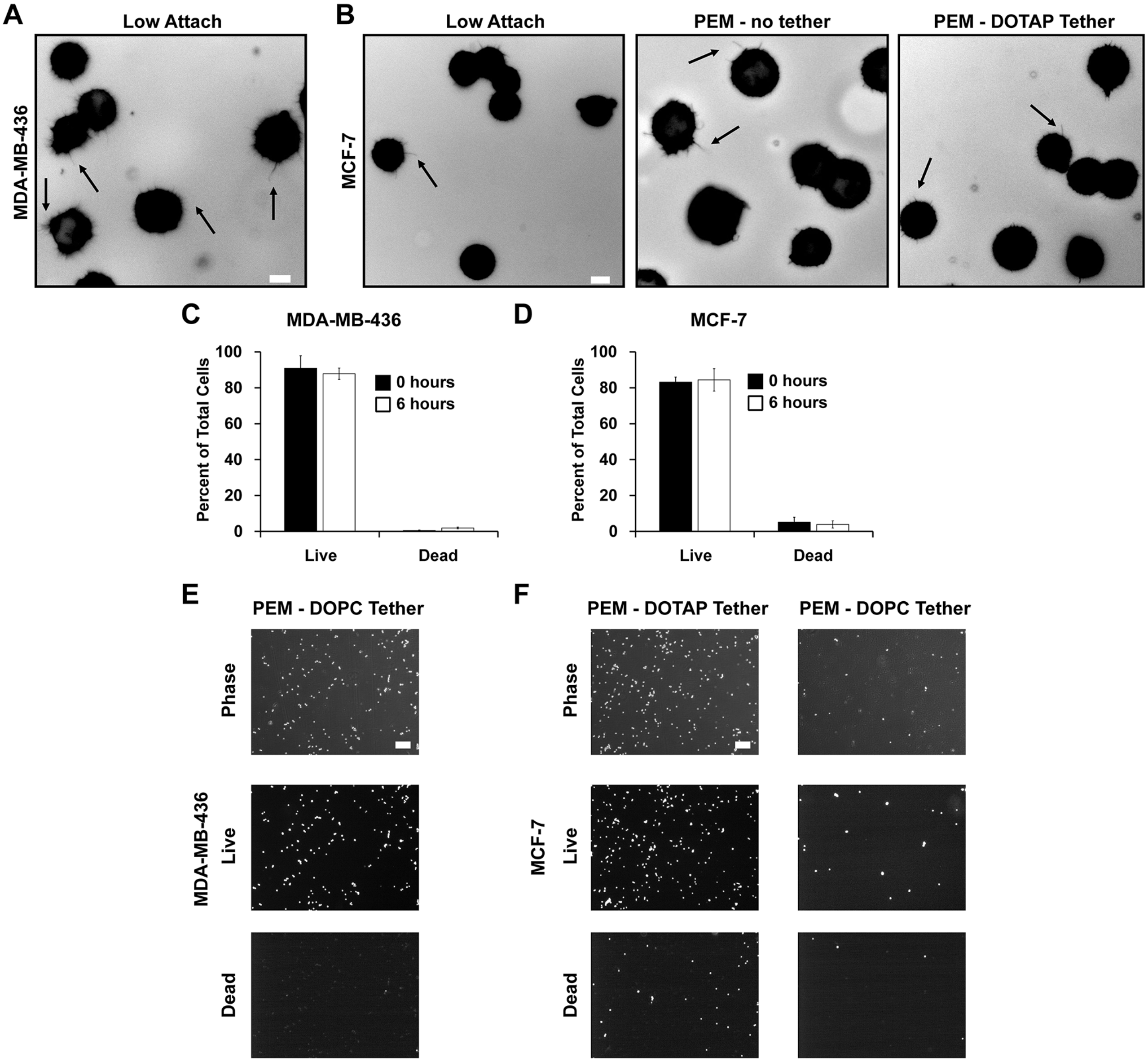

Supplementary Figure S6: Lipid tethering retains microtentacles and does not affect cell viability. A. Representative image of McTNs (arrows) on MDA-MB-436 cells on a low-attach plate at 40x magnification. Scale bar represents 10µm. **B.** Representative images of McTNs (arrows) on MCF-7 cells on a low-attach plate, microfluidic slide with PEM-no tether, and microfluidic slide with PEM-DOTAP tether at 40x magnification. Scale bar represents 10µM. Viability of **C.** MDA-MB-436 and **D.** MCF-7 cells calculated at 0 and 6 hrs after seeding on microfluidic slides with PEM-DOPC tether. Green fluorescence (live) and red fluorescence (dead) positive cells were quantified for each and divided by total cell number to calculate percent of live and dead cells, respectively, using CellProfiler. Data represents mean cell viability from three independent experiments (mean ± SEM). **E.** Representative images of MCF-7 cells seeded on microfluidic slides with PEM-DOTAP tether for 6 hours. Phase contrast, live (green fluorescence), and dead (red fluorescence) images taken at 4x magnification. Scale bar represents 200µm.

Supplementary Figure S7: Lipid tethering allows for real-time microtentacle imaging and shows effects of drugs on microtentacle dynamics without drift. Representative movies at a 10 second frame rate are shown. Each frame is a max intensity projection of five 1µm z-sections. **A.** McTNs on MDA-MB-436 cells seeded on microfluidic slide with PEM-no tether **B.** McTNs on MDA-MB-436 cells seeded on microfluidic slide with PEM-DOTAP tether. **C**. McTNs on MDA-MB-436 cells seeded on microfluidic slide with PEM-DOTAP tether treated with vehicle control. **D.** McTNs on MDA-MB-436 cells seeded on microfluidic slide with PEM-DOTAP tether treated with 5µM colchicine for 15 mins. **E.** McTNs on MDA-MB-436 cells seeded on microfluidic slide with PEM-DOTAP tether treated with 1µg/ml paclitaxel for 120 mins.

(See Supplementary File 1)
