# Supplementary material for: Lipid tethering of breast tumor cells enables real-time imaging of free-floating cell dynamics and drug response
Source: Oncotarget. 2016 Feb 8;7(9):10486–97. doi: 10.18632/oncotarget.7251 (PMC4891134; doi:10.18632/oncotarget.7251)

Supplemental 7: Lipid tethering allows for real-time microtentacle imaging and shows effects of drugs on microtentacle dynamics without drift.

Representative movies taken at a 10 second frame rate are shown. Each frame is a max intensity projection of five 1μm z-sections.

(A) McTNs on MDA-MB-436 cells seeded on microfluidic slide with PEM-no tether

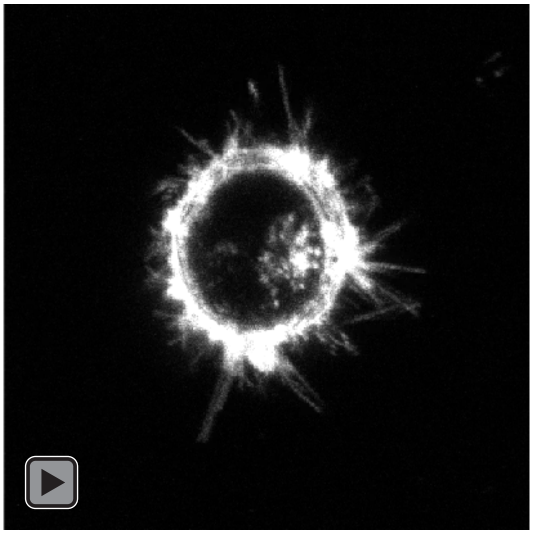

(B) McTNs on MDA-MB-436 cells seeded on microfluidic slide with PEM-DOTAP tether.

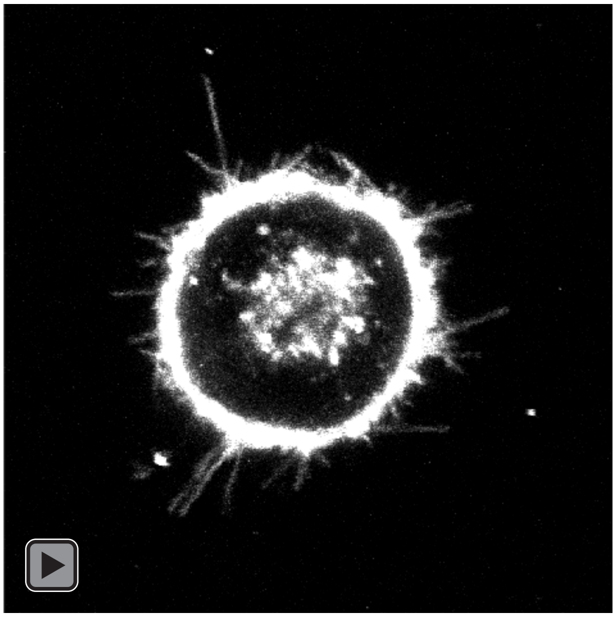

(C) McTNs on MDA-MB-436 cells seeded on microfluidic slide with PEM-DOTAP tether treated with vehicle control.

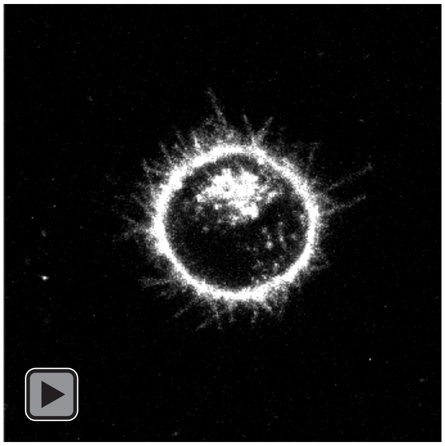

(D) McTNs on MDA-MB-436 cells seeded on microfluidic slide with PEM-DOTAP tether treated with 5µM colchicine for 15 mins.

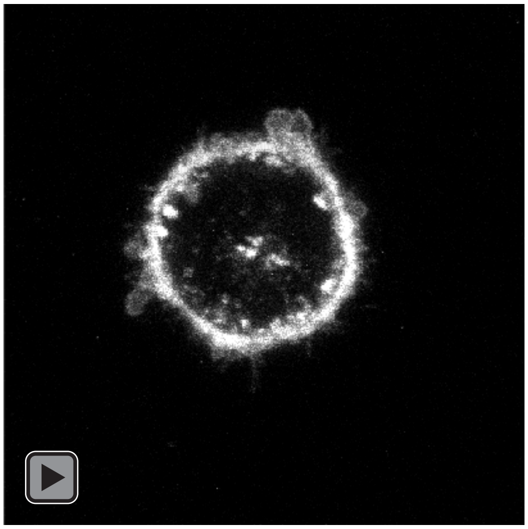

(E) McTNs on MDA-MB-436 cells seeded on microfluidic slide with PEM-DOTAP tether treated with 1µg/ml paclitaxel for 120 mins.

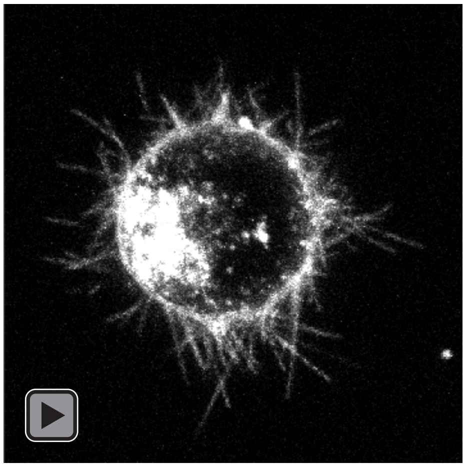

Supplement: Supplementary file 2 [file oncotarget-07-10486-s002.pdf]
